# Renal artery stenosis due to neurofibromatosis

**DOI:** 10.4103/0974-2069.58323

**Published:** 2009

**Authors:** Ishwar Chandra Malav, S S Kothari

**Affiliations:** Department of Cardiology, Cardiothoracic Centre, All India Institute of Medical Sciences, New Delhi, India

**Keywords:** Neurofibromatosis type 1, renovascular hypertension, percutaneous transluminal renal angioplasty

## Abstract

A 4-year-old boy with hypertension due to renal artery stenosis and neurofibromatosis type 1 is presented for its rarity. Renal artery stenosis due to neurofibromatosis is underrecognized and may masquerade Takayasu’s arteritis in Asian children.

## INTRODUCTION

Hypertension in children is frequently underdiagnosed. Severe secondary hypertension may result from renal artery stenosis apart from other causes. The most common cause of renal artery stenosis in children is Takayasu’s arteritis, which may be angiographically indistinguishable from renal artery stenosis due to neurofibromatosis Type 1 (NF1). NF1, an autosomal dominant hamartomatosis, affects 1 in 3000 people.[1] The incidence of hypertension in patients with NF1 is approximately 1% and is mostly due to renal artery stenosis in children.[2] Early diagnosis of renal artery stenosis is warranted as curative treatment in the form of renal angioplasty or surgical repair can prevent the adverse consequences of hypertension.

## CASE REPORT

A 4-year-old boy presented with a 6 month history of breathlessness, cough, and headache. There was no history of oliguria, hematuria, lower limb pain, visual disturbances, or seizures. Early infancy was uneventful with normal development. Family history was notable for the absence of any history of hypertension or neurofibromatosis.

**Figure 1:**
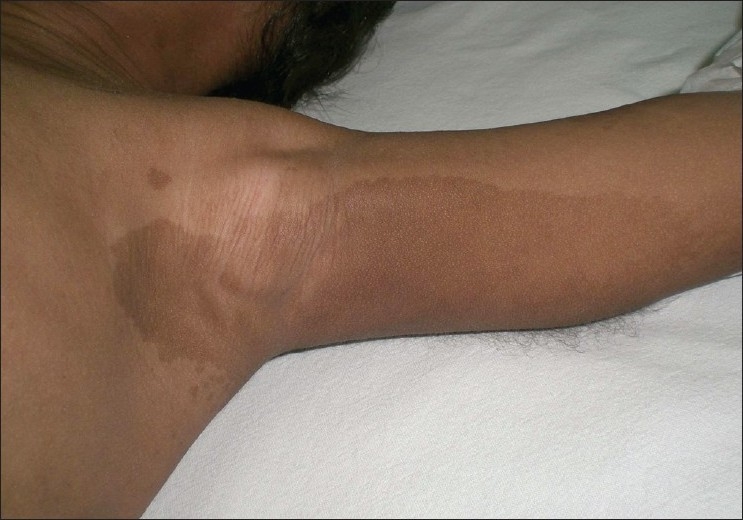
Axillary freckling, café-au-lait spots and neurofibroma

**Figure 2:**
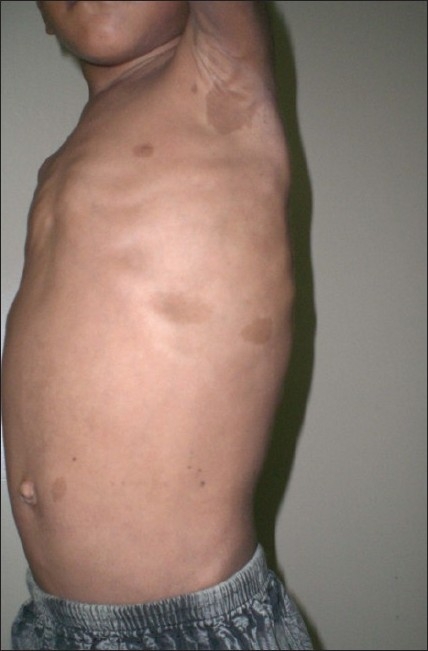
Multiple café-au-lait spots

A clinical examination revealed axillary freckles, multiple café au lait spots over the trunk, and axilla and firm skin swellings in the left axilla and over the spine [Figures 1 and 2]. He was found to have severe hypertension and mild congestive heart failure. His pulse rate was 110/ minute. All peripheral pulses were palpable and there was no radiofemoral delay. Blood pressure was recorded in all four limbs: right arm = 160/102 mmHg, left arm = 158/98, right leg = 164/100 and left leg = 164/100 (99^th^ percentile for age, gender, and height). A systemic examination revealed bibasilar crepitations, cardiomegaly, left ventricular third heart sound, and hepatomegaly. Grade I hypertensive retinopathy was present on fundus examination.

Laboratory parameters including hemogram, renal and liver function tests, urine examination, and serum catecholamines were within normal limits. An electrocardiogram revealed left ventricular hypertrophy with strain pattern and left atrial enlargement. Cardiomegaly (cardiothoracic ratio 70%) and moderate pulmonary venous hypertension was noted on chest x-ray. An echocardiogram demonstrated concentric left ventricular hypertrophy, global hypokinesia, severe left ventricular dysfunction with ejection fraction of 20%, and normal aortic arch. Ultrasound examination of the abdomen was normal. Both kidneys were of normal size (right kidney was 6 cm in length and the left kidney was 6.5 cm in length). A computed tomography (CT) scan of the chest showed a soft tissue mass in the posterior mediastinum and widening of the intervertebral foramina due to neurofibroma at the C6-C7 and C7-T1 level. Abdominal CT scan did not show any adrenal or other abdominal mass. A CT angiography of the renal vessels showed left renal artery stenosis, which was ostial in location. There was no abdominal coarctation of aorta. The final diagnosis of NF1 with left renal artery stenosis, secondary hypertension with severe left ventricular dysfunction, and congestive heart failure was made.

**Figure 3:**
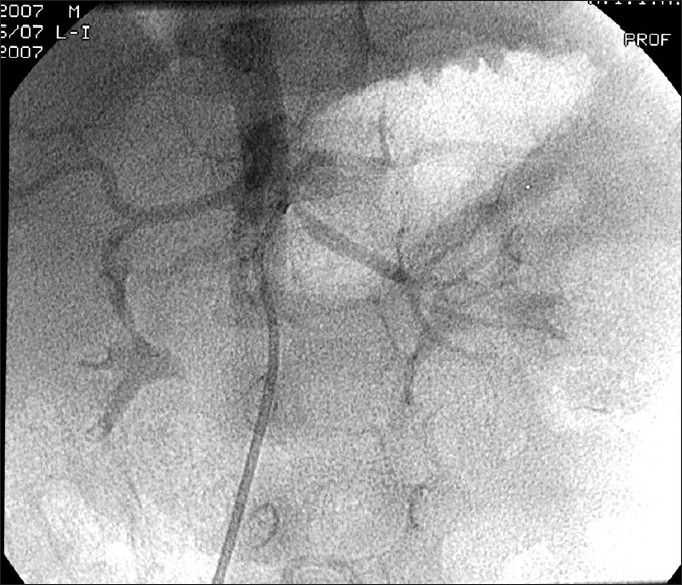
Renal angiogram showing tight ostial stenosis of left renal artery

**Figure 4:**
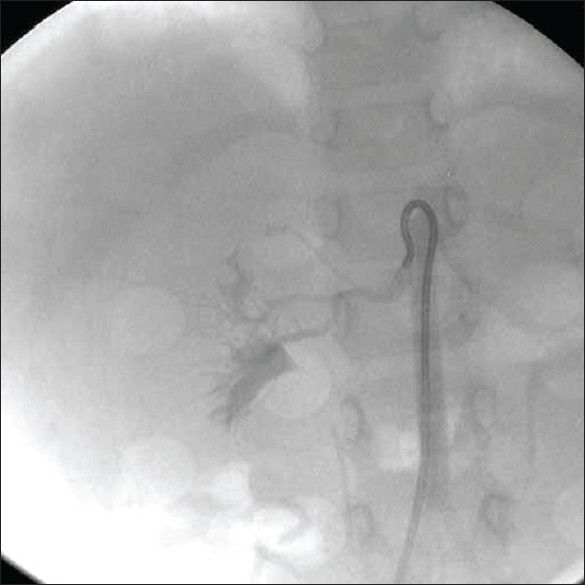
Selective right renal angiogram showing normal right renal artery

The patient was treated with intravenous nitroglycerine, diuretics, and amlodepine. After the initial stabilization, he underwent percutaneous transluminal renal angioplasty (PTRA). Renal angiography revealed left renal artery stenosis at the ostium [Figure 3]. The right renal artery was normal [Figure 4]. PTRA of the left renal artery was done using a 2.5 × 10 mm balloon with good end result. A ngiotensin-converting enzyme (ACE) inhibitor captopril was added to the treatment after PTRA. Thereafter, the patient improved with a significant decline in blood pressure and resolution of congestive cardiac failure. At the follow-up, there was good control of blood pressure.

## DISCUSSION

Renovascular hypertension is an important cause of secondary hypertension in children.[3] It is responsible for 3.0% to 8.5% of pediatric hypertension. The most common cause of renovascular hypertension in children reported in western literature is fibromuscular dysplasia (FMD) and midaortic syndrome.[4] However, in Asian children, Takayasu’s arteritis is the most important cause.[5,6] Renal artery stenosis may also occur in association with NF1, Williams syndrome, Marfan syndrome, congenital Rubella syndrome, Kawasaki disease, and Crohn’s disease.[7]

NF1 typically presents with café au lait spots, multiple neurofibroma, axillary freckling, and ocular Lisch nodules. Hypertension is present in 1% of NF1 patients and is significantly associated with mortality and morbidity. The most frequent cause of hypertension in a child with neurofibromatosis is renal artery stenosis with the median age at diagnosis of hypertension being 5 years.[2] Phaeochromocytoma, coarctation of abdominal aorta, and paraganglioma represent other important causes. Renal artery stenosis in NF1 is mostly ostial in location as was seen in our case. In this regard, it resembles Takayasu’s arteritis. The angiographic picture of renal artery stenosis may be strikingly similar in Takayasu’s arteritis and NF1. Histologically, larger vessels (aorta, proximal renal arteries, and carotid arteries) are surrounded by neurofibromatous or ganglioneuromatous tissue in the adventitia and there is intimal proliferation, thinning of the media, and fragmentation of the elastic tissue.[8] In contrast to fibromuscular dysplasia, which affects main renal arteries, renal artery stenosis in NF1 may involve the intrarenal vessels as well.

Treatment modalities in renovascular hypertension secondary to NF1 involve a combination of drug therapy, PTRA, and surgery. A review of 16 patients of NF1 and renal artery stenosis who underwent PTRA showed a 33% success rate of PTRA for primary stenoses. The success rate was higher (67%) in post-surgery residual stenoses. [2] Because of the absence of major complications and no adverse effect on subsequent vascular reconstruction, PTRA should be considered as first-line treatment for renal artery stenosis in NF1 patients when clinically indicated. In comparison, results of PTRA are better in Takayasu’s arteritis. A technical success of up to 95% has been reported in 2 large studies of PTRA for renal artery stenosis due to Takayasu’s arteritis.[9,10] Restenosis occurs in 20-34% of patients, but repeat PTRA produces significant benefit. In both NF1 and Takayasu’s arteritis, the presence of ostial stenosis and long segment disease predisposes to restenosis. Surgical treatment of renal artery stenosis by reimplanting the renal artery on the aorta, or bypassing the obstruction using the saphenous vein or splenic artery have been reported with good results.[12] Although restenosis after PTRA is not uncommon, there are several advantages of PTRA over surgery. Reduced morbidity from lack of a surgical incision and reduced hospital stay are desirable at all ages. Also, PTRA does not interfere with subsequent surgery if it is needed.

In addition to renal artery stenosis, vascular lesions in NF1 may involve mesenteric vessels, aorta, and cerebral vessels. Vasculopathy of NF1 tends to be progressive and is one of the leading causes of death in this population. [11] Careful long-term follow-up is therefore required.

## CONCLUSIONS

NF1 may present with hypertension due to renal artery stenosis in children. A wider appreciation of this entity is warranted for early diagnosis and appropriate and prompt treatment.
